# Prognostic impact and diagnostic value of invasively derived hemodynamic measures in patients with severe aortic stenosis undergoing TAVI

**DOI:** 10.1007/s00392-023-02154-y

**Published:** 2023-01-19

**Authors:** David Grundmann, Alina Goßling, Lennard Schmidt, Lisa Voigtlaender, Sebastian Ludwig, Matthias Linder, Lara Waldschmidt, Till Demal, Oliver D. Bhadra, Andreas Schaefer, Hermann Reichenspurner, Stefan Blankenberg, Lenard Conradi, Dirk Westermann, Moritz Seiffert, Niklas Schofer

**Affiliations:** 1 Department of Cardiology, University Heart and Vascular Center Hamburg, University Medical Center Hamburg-Eppendorf, Martinistraße 52, 20246 Hamburg, Germany; 2 German Center for Cardiovascular Research (DZHK), Partner Site Hamburg/Lübeck/Kiel, Hamburg, Germany; 3 Department of Cardiovascular Surgery, University Heart and Vascular Center Hamburg, University Medical Center Hamburg-Eppendorf, Martinistraße 52, 20246 Hamburg, Germany; 4 Department of Cardiology and Angiology, University Heart Center, Faculty of Medicine, University of Freiburg, Hugstetter Straße 55, 79106 Freiburg, Germany

**Keywords:** TAVI, Hemodynamic evaluation, Ejection time, Acceleration time, TLVAO

## Abstract

**Background:**

Ejection time (ET), acceleration time (AT) and time between left ventricular and aortic systolic pressure peaks (T-LVAo) might be of diagnostic and prognostic use in patients with aortic stenosis (AS) undergoing transcatheter aortic valve implantation (TAVI).

**Aim:**

We aimed to assess the diagnostic value and prognostic impact of invasively measured ET, AT, and T-LVAo in patients undergoing TAVI.

**Methods:**

A total of 1274 patients received invasive measurement of ET, AT and T-LVAo prior to TAVI. Anatomic AS severity was assessed by CT-derived aortic valve calcification density (AVC_d_). Impact on all-cause mortality was retrospectively analyzed.

**Results:**

In multivariable linear regression, T-LVAo showed the strongest correlation with AVC_d_. No prognostic impact of T-LVAo was found according to uni- and multivariable analyses. In contrast, using an individual *C*-statistic derived cutoff (*C*_D_), patients with ET or AT ≥ *C*_D_ showed lower mortality rates compared to patients with ET or AT < *C*_D_ (1-year mortality: ET ≥ vs. < *C*_D_: 15.01vs. 33.1%, AT ≥ vs < *C*_D_ 16.3 vs. 26.5%, *p < *0.001). Moreover, multivariable analysis identified ET ≥ *C*_D_ (HR 0.61 [95% CI 0.43–0.87; *p < *0.007]) to be associated with beneficial outcome after TAVI, independent from clinical risk factors and echocardiography-derived parameters.

**Conclusion:**

Among the studied hemodynamic parameters T-LVAo provides the highest diagnostic value, whereas ET is an outcome predictor beyond clinical risk factors and echocardiographic parameters in AS patients following TAVI. These parameters could be of considerable use in diagnostic evaluation and risk assessment of patients scheduled for TAVI.

**Graphical abstract:**

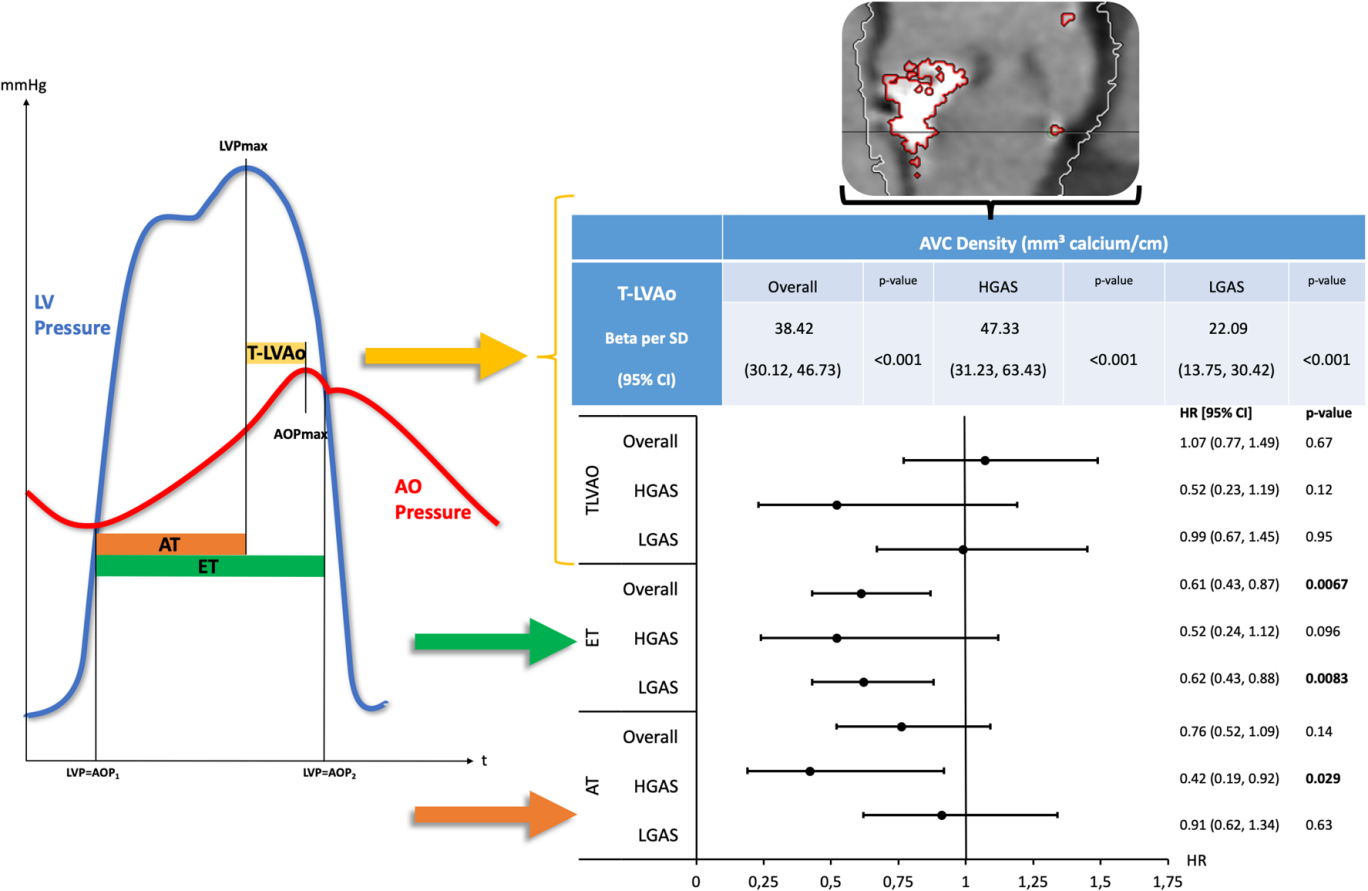

T-LVAo (yellow): defined as time between left ventricular and aortic systolic pressure peaks. ET (green): Ejection Time defined as time from the start to flow end. AT (orange): Acceleration time defined as time from the start to the peak flow. AOP: aortic pressure, AVC: aortic valve calcification, CI: confidence interval, HGAS: high-gradient aortic stenosis, LGAS: low-gradient aortic stenosis, LVP: left ventricular pressure, SD: standard deviation.

**Supplementary Information:**

The online version contains supplementary material available at 10.1007/s00392-023-02154-y.

## Introduction

As aortic stenosis (AS) progresses and effective orifice area (EOA) decreases, significant changes in transvalvular flow patterns become apparent. These changes include a prolongation of time from start to peak flow, i.e. acceleration time (AT), as well as an increase of ejection time (ET), defined as the overall time of transvalvular blood flow. Both variables, as assessed by echocardiography, have been correlated with AS severity and are associated with poor prognosis in AS patients under medical management [1–4].

Furthermore, a recent publication showed that time between invasively measured left ventricular and aortic systolic pressure peaks (T-LVAo), might serve as another hemodynamic parameter correlating with anatomic AS severity as assessed by CT based quantification of aortic valve calcification [5].

So far, the prognostic value of these parameters in aortic valve replacement, remains unknown. Moreover, correlation of T-LVAo with AS-severity has not been validated in a larger patient cohort.

Thus, in this analysis we aimed to assess the prognostic and diagnostic impact of T-LVAo, ET and AT as assessed by invasive measurements in patients undergoing transcatheter aortic valve implantation (TAVI) for severe AS.

## Methods

### Patient population

From January 2012 until August 2019, 2720 consecutive patients who underwent TAVI for severe AS at the University Heart and Vascular Center Hamburg were included in this single-centre retrospective analysis. 1,388 patients were excluded from analysis due to missing data on invasive and echocardiographic hemodynamic evaluation, 58 patients due to valve-in-valve procedures, leaving a total of 1,274 patients for analyses. The study population was stratified into two groups according to Pmean: low-gradient aortic stenosis (< 40 mmHg, EOA ≤ 1cm^2^; LGAS; *n = *787) and high-gradient aortic stenosis (≥ 40 mmHg, EOA ≤ 1cm^2^; HGAS; *n = *487).

### Clinical outcomes and endpoint definitions

All cases were reviewed by the local heart team and agreed to be eligible for TAVI. Periprocedural results and clinical outcomes were consecutively assessed according to the updated Valve Academic Research Consortium definitions [6].

### Computed tomography assessment

Routine contrast-enhanced multi-detector computed tomography (MDCT) was performed during pre-TAVI workup, as described before [7]. Dimensions of the aortic annulus and root and calcification of the aortic valve complex were assessed with the 3-Mensio Structural Heart Software V9.1, (Pie Medical Imaging, Maastricht, Netherlands). Anatomic AS severity was assessed according to MSCT-derived aortic valve calcification density (AVC_d_) defined as calcium volume of AVC and LVOT per annulus area as described previously [8]. Quantification in contrast-enhanced MDCT images is depicted in Supplementary Fig. 1.

### Echocardiographic evaluation

All patients received transthoracic (TTE) and/or transesophageal echocardiographic (TOE) evaluation prior to TAVI according to current guidelines. Measured values included left-ventricular ejection fraction (LVEF), EOA, which was determined using the continuity equation, mean atrioventricular pressure gradient (Pmean) and Stroke volume index (SVI). Velocities were assessed in numerous views using continuous-wave Doppler, with pressures being automatically derived utilizing the Bernoulli equation. AS was defined as EOA < 1.0 cm^2^. According to Pmean AS types LGAS (< 40 mmHg) and HGAS (≥ 40 mmHg) were characterized.

### Hemodynamic evaluation

**Fig. 1:**
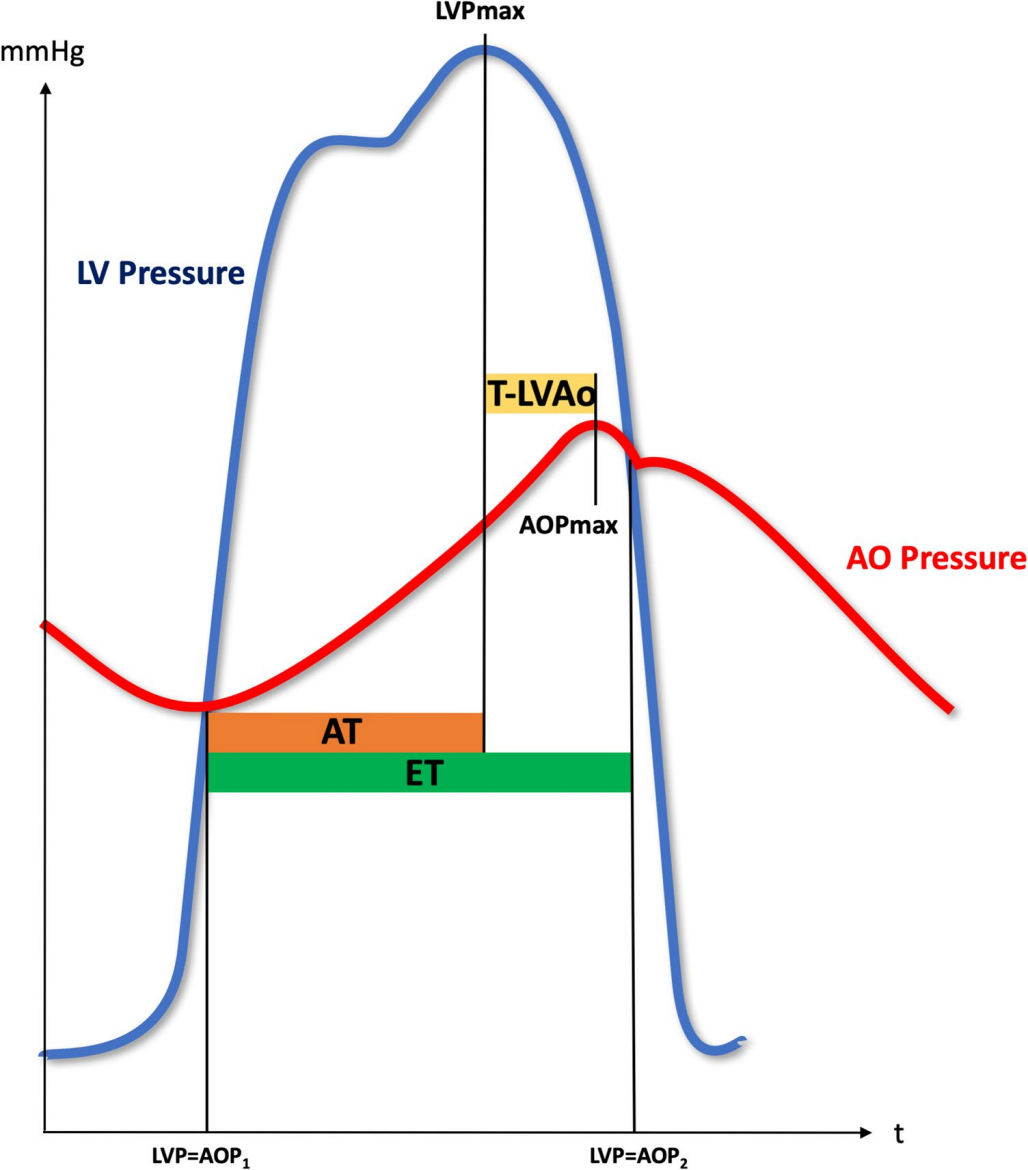
Invasively derived hemodynamic parameters. T-LVAo (yellow): defined as time between left ventricular and aortic systolic pressure peaks. ET (green): ejection time defined as time from the start to flow end. AT (orange): acceleration rime defined as time from the start to the peak flow. *AOP* aortic pressure, *LVP* left ventricular pressure

Invasive hemodynamic parameters were assessed by simultaneous pressure measurements in left ventricle and aorta during TAVI prior to valve deployment using 2 fluid-filled catheters. All hemodynamic parameters were calculated offline using a dedicated software (Schwarzer Cardiotek) by a trained analyst. T-LVAo was defined as time between left ventricular and aortic systolic pressure peaks, ET as time from the start to end of left ventricular flow and AT as time from the start to peak of ventricular flow. Assessment of all invasively hemodynamic parameters is shown in Fig. 1. The intra- and interobserver intraclass-correlation coefficient for hemodynamic parameter measurements in 50 randomly selected patients were high (*κ*-index = 0.994 [95% CI 0.989–0.997; *p = *0.0001 and 0.996 [95% CI 0.993–0.998; *p = *0.0001] respectively) indicating good reliability.

### Statistical analysis

Patients were divided into LGAS and HGAS. Binary variables were shown as absolute numbers or percentages and were compared using the *χ*^2^ test. Continuous variables were shown as median (25th, 75th percentile) and were compared using the Mann–Whitney test.

Multiple linear regressions were used to assess the correlation between hemodynamic parameters individually and AVC_d_. A multivariable model with all three parameters were designed. Correlation is depicted as Beta per standard deviation (SD).

Median follow-up time and event rates were calculated by the Kaplan–Meier potential follow-up estimator. Survival curves for all-cause mortality were produced using the Kaplan–Meier method. The log-rank test was used to test for survival curve differences.

Mortality predictors were assessed using univariable and multivariable Cox regression analyses through a best performing selection process. The following variables were used for this process: age, male sex, body mass index (BMI) in categories, prior myocardial infarction, prior stroke, LVEF categories, log transformed AVC_d_, pulmonary hypertension, diabetes and treatment, prior atrial fibrillation, chronic obstructive pulmonary disease (COPD), impaired kidney function (GFR < 60 mL/min), Pmean, SVI and invasively determined heart rate. Variables that showed *p* values < 0.25 in the univariable Cox regression analyses were used in a forward selection process based on AIC. T-LVAo, ET and AT, respectively, were forced into the final model.

All *p* values had a significance threshold of < 0.05. Statistical analyses were performed using R version 4.0.3 (R Foundation for Statistical Computing).

## Results

### Baseline characteristics

Patients with LGAS were more often male and showed higher prevalence of NYHA IV, prior myocardial infarction, prior cardiac surgery, insulin-dependent diabetes, coronary artery disease (CAD), peripheral artery disease (PAD), atrial fibrillationand reduced kidney function compared to those with HGAS, resulting in a higher predicted operative risk for mortality according to EuroSCORE II. Echocardiography at baseline showed lower mean gradient, SVI and higher prevalence of LVEF < 30% in LGAS-Patients. Of those 43.7% presented with Normal-Flow-LGAS (NF-LGAS), 33.3% with Low-EF-LGAS (LEF-LGAS) and 23.0% with Paradoxical-Low-Flow-LGAS (PLF-LGAS). Moreover, patients with LGAS presented with lower AVC_d_ as well as lower T-LVAo and ET. Complete baseline and diagnostic parameters are shown in Tables 1 and 2, procedural characteristics in Supplementary Table 1.

**Table 1:** Baseline characteristics

	All (*N = *1274)	HGAS (*N = *487)	LGAS (*N = *787)	*p* value
Sex (male) (%)	571 (44.8)	182 (37.4)	389 (49.4)	** < 0.001**
Age (years)	81.4 (77.1, 84.8)	81.6 (77.3, 85.2)	81.3 (76.9, 84.7)	0.22
STS PROM (%)	3.8 (2.5, 5.6)	3.7 (2.4, 5.5)	3.9 (2.6, 5.7)	0.061
Logistic EuroSCORE II (%)	3.8 (2.3, 6.2)	3.2 (2.1, 5.1)	4.1 (2.4, 7.5)	** < 0.001**
NYHA III (%)	903 (72.2)	350 (72.9)	553 (71.8)	0.72
NYHA IV (%)	140 (11.2)	39 (8.1)	101 (13.1)	**0.0085**
Prior myocardial infarction (%)	173 (13.6)	45 (9.2)	128 (16.3)	** < 0.001**
Diabetes: insulin (%)	187 (14.7)	49 (10.1)	138 (17.5)	** < 0.001**
Previous cardiac surgery (%)	149 (11.7)	27 (5.5)	122 (15.5)	** < 0.001**
Hypertension (%)	1117 (87.7)	431 (88.5)	686 (87.2)	0.54
Pulmonary hypertension: PAP syst > 55 mmHg (%)	159 (12.5)	72 (14.8)	87 (11.1)	0.061
CAD (%)	801 (63.4)	261 (54.1)	540 (69.1)	** < 0.001**
PAD (%)	330 (25.9)	107 (22.0)	223 (28.3)	**0.015**
COPD (%)	202 (15.9)	71 (14.6)	131 (16.6)	0.37
Atrial fibrillation (%)	499 (39.2)	147 (30.2)	352 (44.7)	** < 0.001**
Prior stroke (%)	183 (14.4)	61 (12.5)	122 (15.5)	0.16
Anemia (Hb < 11) (%)	360 (28.3)	164 (33.7)	196 (25.0)	** < 0.001**
GFR (CKD-EPI) (all) (mL/min/1.73m^2^)	57.8 (41.3, 74.4)	61.3 (43.8, 77.8)	56.0 (39.9, 71.5)	** < 0.001**

Values are *n* (%) or median (25th, 75th percentile)

Bold indicates *p* < 0.05

*CAD* coronary artery disease, *COPD* chronic obstructive pulmonary disease, *GFR* glomerular filtration rate, *HGAS* high gradient aortic stenosis, *LGAS* low gradient aortic stenosis, *NYHA* New York Heart Association, *PAD* peripheral artery disease, *PAP* pulmonary artery pressure, *STS-PROM* Society of Thoracic Surgeons Predicted Risk of Mortality

**Table 2:** Diagnostic parameters

	All (*N = *1274)	HGAS (*N = *487)	LGAS (*N = *787)	*p* value
Echocardiography				
LVEF < 30% (%)	140 (11.0)	23 (4.7)	117 (14.9)	** < 0.001**
EOA per BSA (cm^2^/m^2^)	0.4 (0.3, 0.5)	0.4 (0.3, 0.4)	0.4 (0.4, 0.5)	**< 0.001**
AVA (baseline)	2.0 (1.6, 2.4)	2.0 (1.5, 2.4)	2.0 (1.6, 2.4)	0.41
EOA (cm^2^)	0.8 (0.6, 0.9)	0.7 (0.6, 0.8)	0.8 (0.7, 0.9)	**< 0.001**
*p* mean (mmHg)	34.0 (25.0, 46.0)	49.0 (44.0, 57.8)	27.0 (21.0, 33.0)	**< 0.001**
HGAS (%)	487 (38.2)	487 (100)	0 (0)	
LGAS (%)	787 (61.8)		787 (100)	
PLF-LGAS (%)	180 (14.2)		180 (23.0)	
LEF-LGAS (%)	261 (20.6)		261 (33.3)	
NF-LG AS (%)	344 (27.0)		344 (43.7)	
Stroke volume index (mL/m^2^)	36.3 (29.0, 44.8)	41.6 (34.4, 48.4)	33.2 (27.3, 40.4)	** < 0.001**
Severe aortic regurgitation (%)	20 (1.6)	10 (2.1)	10 (1.3)	0.40
Severe mitral regurgitation (%)	93 (7.4)	26 (5.4)	67 (8.6)	**0.044**
Severe tricuspid regurgitation (%)	75 (6.0)	19 (3.9)	56 (7.3)	**0.020**
MDCT				
AVC total (pre)	474.1 (259.1, 812.9)	741.4 (467.6, 1063.9)	366.2 (218.6, 628.8)	**< 0.001**
AVC density (mm^3^ calcium/cm^2^)	106.0 (58.8, 175.6)	170.6 (113.4, 244.8)	81.0 (48.4, 136.5)	**< 0.001**
Annulus area (pre)	464.8 (401.9, 531.3)	437.3 (385.4, 509.3)	479.6 (420.8, 540.9)	**< 0.001**
Hemodynamic evaluation				
Peak to peak gradient (pre) (mmHg)	41.0 (27.0, 59.3)	61.0 (49.0, 76.8)	31.0 (21.0, 43.0)	**< 0.001**
T-LVAo (ms)	70.0 (46.0, 96.2)	84.0 (62.0, 112.0)	60.0 (40.0, 88.0)	**< 0.001**
Ejection time (ms)	308.0 (277.8, 336.0)	324.0 (298.0, 350.0)	296.0 (266.0, 326.0)	**< 0.001**
Acceleration time (ms)	180.0 (146.0, 206.0)	182.0 (148.3, 208.0)	178.0 (144.0, 204.0)	0.087
Heart rate (invasively derived) (mmHg)	69.0 (60.0, 79.0)	67.0 (59.0, 78.0)	69.0 (60.0, 80.0)	0.079

Values are *n* (%) or median (25th, 75th percentile)

Bold indicates *p* < 0.05

*AVA* aortic valve area, *AVC* aortic valve calcification, *BSA* body surface area, *EOA* effective orifice area, *HGAS* high gradient aortic stenosis, *LVEF* left-ventricular ejection fraction, *LGAS* low gradient aortic stenosis, *LEF-LG AS* low-ef-low-gradient aortic stenosis, *MDCT* multi-detector computed tomography, *NF-LG AS* normal-flow-low-gradient aortic stenosis, *PLF-LG AS* paradoxical low-flow-low-gradient aortic stenosis, *SVI* stroke volume index, *T-LVAo* time between left ventricular and aortic systolic pressure peaks

### Correlation of hemodynamic parameters with AS severity

Table 3 shows correlation of T-LVAo, ET, and AT with AS severity as assessed by AVC_d_ according to multiple linear regression analysis for all patients as well as LGAS and HGAS patients, separately. Prolonged T-LVAo was significantly associated with higher AVC_d_ in both HGAS and LGAS-patients. In contrast, ET did not show any significant correlation with AVC_d_. Prolonged AT was associated with elevated AVC_d_ overall and in patients with HGAS. However, this correlation was weaker compared to T-LVAo and there was no significant correlation of AT with AVC_d_ among LGAS patients. Correlation of the investigational parameters with with echocardiographic markers of AS severity, i.e. EOA and Pmean, is given in supplementary Table 2. Again, T-LVAo provided the stongest inverse correlation with EOA and the second strongest correlation with Pmean.

**Table 3:** Multivariable linear regression of invasively derived parameters for correlation with AS severity

	Overall AVC density, (mm^3^ calcium/cm)	*p* value	HGAS AVC density, (mm^3^ calcium/cm)	*p* value	LGAS AVC density, (mm^3^ calcium/cm)	*p* value
T-LVAo						
Beta per SD (95% CI)	38.42 (30.12, 46.73)	** < 0.001**	47.33 (31.23, 63.43)	< **0.001**	22.09 (13.75, 30.42)	** < 0.001**
SD	37.64		36.55		36.57	
ET						
Beta per SD (95% CI)	5.58 (− 3.02, 14.19)	0.20	− 12.65 (− 30.04, 4.73)	0.15	− 0.15 (− 8.50, 8.19)	0.97
SD	49.61		46.52		48.02	
AT						
Beta per SD (95% CI)	15.29 (5.24, 25.35)	**0.0029**	28.08 (8.07, 48.09)	**0.0061**	9.32 (− 0.86, 19.50)	0.073
SD	45.67		43.86		46.53	

Bold indicates *p* < 0.05

*AT* acceleration time, *AVC* aortic valve calcification, *CI* confidence interval, *EF* ejection fraction, *ET* ejection time, *HGAS* high gradient aortic stenosis, *LVEF* left-ventricular ejection fraction, *SD* standard deviation, *T-LVAo* time between left ventricular and aortic systolic pressure peaks

### Survival analysis

Median follow-up-time overall was 1 year with 65 events at 30 days, 189 events at 1 year and 247 events at 3 years.

**Fig. 2:**
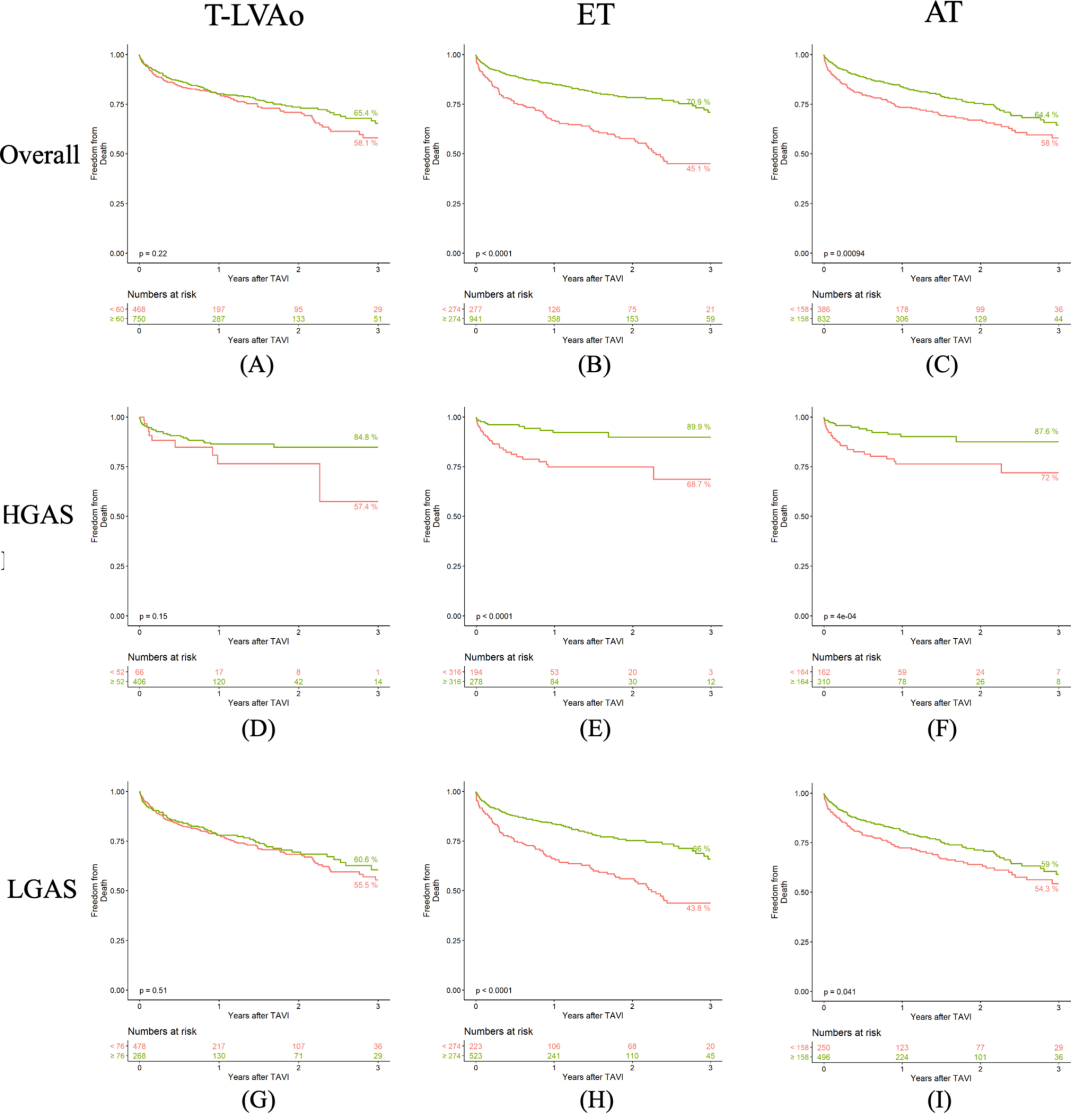
Kaplan–Meier survival up to 3 years after TAVI. Survival according to c-statistic determined cutoffs for invasively derived parameters for all patients (3A T-LVAo < 60 vs. ≥ 60 ms, 3B ET < 274 vs. ≥ 274 ms, 3C AT < 158 vs. ≥ 158 ms), patients with HGAS- (3D T-LVAo < 52 vs. ≥ 52 ms, 3E ET < 316 vs. ≥ 316 ms, 3F AT < 164 vs. ≥ 164 ms) as well as LGAS (3G T-LVAo < 76 vs. ≥ 76 ms, 3H ET < 274 vs. ≥ 274 ms, 3I AT < 158 vs. ≥ 158 ms). Numbers at risk are shown beneath respectively. *AT* acceleration time, *ET* ejection time, *HGAS* high-gradient aortic stenosis, *LGAS* low-gradient aortic stenosis, *T-LVAo* time between left ventricular and aortic systolic pressure peaks

The optimal cutoff for death (*C*_D_) was calculated overall and for each AS-group individually according to *C*-statistics for each hemodynamic parameter (*C*_D_ for all patients for T-LVAo 60 ms, *C*_D_ for ET 274 ms, *C*_D_ for AT 158 ms). Survival according to T-LVAo ≥ vs. < *C*_D_ did not differ significantly in any group (overall survival at 3-years: 65.4 vs. 58.1%, *p*_log-rank_ = 0.22). Patients with ET or AT ≥ *C*_D_ showed lower short and mid-term survival rates compared to patients with ET or AT < *C*_D_ overall (ET ≥ vs. < *C*_D_: overall survival at 3 years: 70.9 vs. 45.1%, *p*_log-rank_ < 0.001; AT ≥ vs < *C*_D_: overall survival at 3 years: 64.4 vs. 58.0%, *p*_log-rank_ < 0.001) as well as in the subgroups of patients with HGAS or LGAS. For detailed survival analysis, see Fig. 2.

### Multivariable analysis

**Fig. 3:**
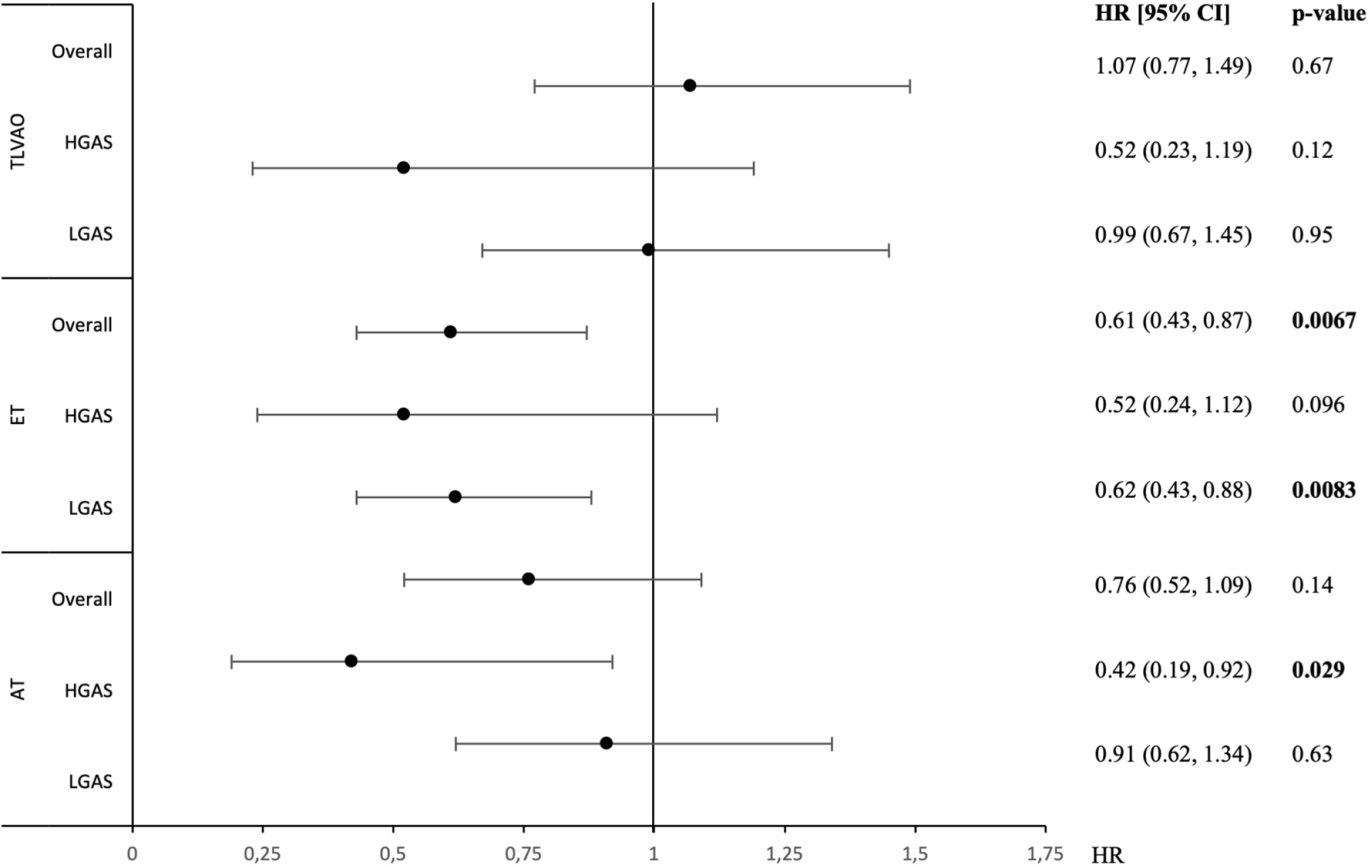
Multivariable analysis for overall mortality after TAVI. Alongside: age, sex, prior MI, prior stroke, EF, AVC_d_, pulmonary hypertension, diabetes on insulin, atrial fibrillation, COPD, GFR < 60, *p* mean, stroke volume index, EOA per BSA, heart rate (invasively derived), respectively. Invasively derived parameters T-LVAo, ET and AT are analyzed as above c-statistic defined cutoff (see Fig. 2). *AT* acceleration time, *CI* confidence interval, *ET* ejection time, *HGAS* high-gradient aortic stenosis, *LGAS* low-gradient aortic stenosis, *T-LVAo* time between left ventricular and aortic systolic pressure peaks

Association of T-LVAo, ET, and AT with all-cause mortality following TAVI as assessed by multivariable analyses is illustrated in Fig. 3. According to multivariable analysis T-LVAo ≥ *C*_D_ showed no significant prognostic impact. In contrast ET ≥ *C*_D_ was, among other variables, associated with beneficial outcome after TAVI overall, as well as in patients with LGAS (HR 0.59 [95% CI 0.43–0.81; *p = *0.001), whereas AT ≥ *C*_D_ showed prognostic impact in patients with HGAS (HR 0.42 [95% CI 0.19–0.92; *p = *0.029), separately and independent from clinical risk factors and echocardiography-derived parameters like LVEF, Pmean or stroke volume index. Further predictors of outcome among the study population were prior MI, impaired kidney function (GFR < 60 ml/min), insulin-dependent diabetes, atrial fibrillation, Pmean and reduced LVEF. Detailed information on multivariable analyses is given in Supplementary Tables 3 to 5.

## Discussion and limitations

This study assessed the diagnostic and prognostic value of invasively derived hemodynamic parameters T-LVAo, ET and AT. The main findings were:

(i) T-LVAo shows very high correlation with anatomical AS severity in HGAS as well as LGAS-patients. (ii) In contrast to T-LVAo, predominantly ET but also AT were associated with significant prognostic impact in patients with HGAS as well as LGAS independent from clinical risk factors and standard echocardiographic parameters.

Identification of severe aortic stenosis according to the continuity equation for effective orifice area and transvalvular gradients in echocardiography is widely established as gold standard [9]. As these variables are flow-dependent, guidelines recommend further diagnostic evaluation such as quantification of aortic valve calcification in MDCT and dobutamine stress echocardiography in case of reduced ejection fraction and low-flow situation. However, other modalities may be needed, especially if results of echocardiography or MDCT are inconclusive. In such cases, current guidelines also suggest invasive evaluation including instantaneous left ventricular and aortic pressure measurements especially when symptoms are typical for severe AS [9]. Besides calculation of aortic valve area using the Gorlin equation, T-LVAo represents a further invasively derived parameter showing robust correlation with anatomical AS severity as recently demonstrated by Sato et al. [5]. In the present study we were able to confirm and validate these results in a large patient cohort, including in HGAS- as well as LGAS-patients and thus demonstrate high diagnostic utility of T-LVAo in cases were alternative diagnostic modalities are needed. In comparison to T-LVAo diagnostic abilities of AT were weaker, however significant, and in case of ET non-existent, which is in accordance with findings of previous studies investigating ET or AT as assessed by echocardiographic means [1–4]. A non-invasive diagnostic approach with imaging modalities like echocardiography is central in the diagnostic workup of AS, especially considering the growing importance of reducing time and invasiveness of the TAVI procedure in line with a simplified approach. The invasive means needed to obtain T-LVAo thus certainly represent a limiting obstacle in routine clinical use, also because all relevant diagnostic parameters should be present prior to the procedure, however can be exploited in cases of inconclusive results in routine diagnostic workup or simultaneous valve-independent indication for invasive coronary angiography.

The most intriguing finding of our study is the association of ET and AT with beneficial outcome following TAVI for severe HGAS and LGAS. To our knowledge this is the first study to investigate ET or AT in patients receiving treatment for severe AS as well as using invasive derivation for doing so. Our results show that prolongation of these parameters, in particular ET, are associated with a reduced mortality up to 3 years after treatment of severe AS with TAVI. At a first glance, these results seem somewhat contradicting as it has been shown that AT and ET, as assessed by echocardiographic means, reflect the LV response to a chronically increased afterload and are actually linked to poor outcome in patients with untreated moderate or severe AS [10, 11]. However, it can be hypothesized that in the presence of severe AS the capacity of the LV to achieve longer ET or AT might serve as an indicator for a higher likelihood of successful reverse remodeling once severe AS is removed by TAVI [12]. In fact, other markers for AS severity who have been associated with poor outcome in untreated patients, such as Pmean or AVC_d_, are also strong predictors of survival in AS patients, who undergo treatment with TAVI [8, 13]. There is undoubtedly debatable comparability of these invasively to their non-invasively derived hemodynamic parameters ET or AT. Future analyses should evaluate ET and AT by strictly non-invasive echocardiographic means and assess their prognostic abilities in patients receiving treatment for severe AS.

Several limitations are inherent to the present study. First, it is a retrospective single-centre study, thus results are only hypothesis-generating. Second, AVC_d_ was derived from contrast-enhanced MDCT-images, which is known to be less accurate compared to non-contrast MDSCT assessment, however, this approach has also been applied in a previous study investigating the diagnostic ability of hemodynamic parameters for patients with severe AS [5, 14]. Third, invasive assessment of hemodynamic parameters is not part of the standard diagnostic workup of AS which limits its application in clinical routine, and which in our view is the main limitation of this study.

## Conclusion

T-LVAo provides very high diagnostic value showing a strong association with AVC_d_, in HGAS as well as LGAS-Patients. Moreover, ET and AT are independent outcome predictors beyond clinical risk factors and standard echocardiographic parameters in AS patients following TAVI. Accordingly, these investigational hemodynamic parameters could be of considerable value in diagnostic evaluation and risk assessment of patients scheduled for TAVI.

## Supplementary Information

Below is the link to the electronic supplementary material.

Supplementary file1 (DOCX 5694 kb)

## Data Availability

The data that support the findings of this study are not publicly available, however can be obtained on request from the corresponding author.

## Abbreviations

AS: Aortic stenosis
AT: Acceleration time
AVC: Aortic valve calcification
AVC_d_: Aortic valve calcification density
CI: Confidence interval
*C*_D_: Cutoff for death
ET: Ejection time
GFR: Glomerular filtration rate
HR: Hazard ratio
LGAS: Low-gradient-aortic-stenosis
LVEF: Left-ventricular ejection fraction
LVOT: Left ventricular outflow tract
MDCT: Multi-detector computed tomography
TAVI: Transcatheter aortic valve implantation
T-LVAo: Time between systolic left-ventricular and aortic pressure peaks
VARC: Valve Academic Research Consortium
