# Supplementary material for: Prognostic impact and diagnostic value of invasively derived hemodynamic measures in patients with severe aortic stenosis undergoing TAVI
Source: Clin Res Cardiol. 2023 Jan 19;112(5):667–76. doi: 10.1007/s00392-023-02154-y (PMC10160203; doi:10.1007/s00392-023-02154-y)
Supplement: Supplementary file 1 — Supplementary file1 (DOCX 5694 kb) [file 392_2023_2154_MOESM1_ESM.docx]

**Supplementary material**

**Supplementary Table 1:** **Procedural characteristics**

|  | All (N=1274) | HGAS (N=487) | LGAS (N=787) | p-value |
| --- | --- | --- | --- | --- |
| Access |  |  |  |  |
| Transfemoral (%) | 1138 (89.3) | 696 (88.4) | 442 (90.8) | 0.23 |
| Transapical (%) | 92 (7.2) | 56 (7.1) | 36 (7.4) | 0.94 |
| Transaxillary (%) | 42 (3.3) | 33 (4.2) | 9 (1.8) | 0.034 |
| Other (%) | 2 (0.2) | 2 (0.3) | 0 (0) | 0.70 |
| THV Type |  |  |  |  |
| Sapien (%) | 4 (0.3) | 0 (0) | 4 (0.8) | 0.042 |
| Sapien XT (%) | 0 (0) | 0 (0) | 0 (0) |  |
| Sapien 3 (%) | 494 (38.8) | 322 (40.9) | 172 (35.3) | 0.053 |
| CoreValve Evolut (%) | 92 (7.2) | 57 (7.2) | 35 (7.2) | 1.00 |
| JenaValve (%) | 18 (1.4) | 12 (1.5) | 6 (1.2) | 0.85 |
| Symetis/Boston Scientific  Acurate (%) | 368 (28.9) | 232 (29.5) | 136 (27.9) | 0.60 |
| Medtronic Engager (%) | 9 (0.7) | 5 (0.6) | 4 (0.8) | 0.97 |
| Lotus (%) | 81 (6.4) | 37 (4.7) | 44 (9.0) | 0.0031 |
| Portico (%) | 115 (9.0) | 63 (8.0) | 52 (10.7) | 0.13 |
| BioValve (%) | 15 (1.2) | 10 (1.3) | 5 (1.0) | 0.90 |
| Centera (%) | 27 (2.1) | 16 (2.0) | 11 (2.3) | 0.94 |
| Allegra (%) | 37 (2.9) | 26 (3.3) | 11 (2.3) | 0.36 |
| THV Type groups |  |  |  |  |
| Balloon-expandable (%) | 498 (39.1) | 176 (36.1) | 322 (40.9) | 0.10 |
| Self-expandable (%) | 695 (54.6) | 267 (54.8) | 428 (54.4) | 0.92 |
| Mechanically-expandable (%) | 81 (6.4) | 44 (9.0) | 37 (4.7) | 0.0031 |
| Anesthesia |  |  |  |  |
| General anesthesia (%) | 325 (25.7) | 200 (25.6) | 125 (25.9) | 0.97 |
| Sedation (%) | 934 (73.9) | 576 (73.8) | 358 (74.1) | 0.94 |
| Other (%) | 5 (0.4) | 5 (0.6) | 0 (0) | 0.19 |
| Procedure time (min) | 80.0 (65.0, 105.0) | 80.0 (65.0, 105.0) | 80.0 (65.0, 105.0) | 0.37 |
| Contrast (mL) | 180.0 (140.0, 221.0) | 175.0 (139.4, 220.0) | 183.0 (146.0, 225.0) | 0.10 |
| Balloon valvuloplasty of native valve (%) | 1009 (79.5) | 595 (75.8) | 414 (85.5) | <0.001 |
| Postdilation (%) | 485 (38.2) | 269 (34.3) | 216 (44.7) | <0.001 |
| Length of stay intensive care unit (days) | 2.6 ± 4.6 | 2.7 ± 5.2 | 2.3 ± 3.4 | 0.090 |
| Length of stay total (days) | 10.8 ± 19.4 | 10.7 ± 15.3 | 11.0 ± 24.7 | 0.79 |

Values are mean ± SD, n (%) or median (25^th^, 75^th^ percentile).

CKD: chronic kidney disease, ESRD: end-stage renal disease, HGAS: high gradient aortic stenosis, ICU: Intensive care unit, LGAS: low gradient aortic stenosis, THV: transcatheter heart valve

**Supplementary Table 2: Multivariable linear regression of invasively derived parameters for correlation with AS severity according to Pmean and EOA**

|  | Pmean (mmHg) | p-value | EOA (cm^2^) | p-value |
| --- | --- | --- | --- | --- |
| T-LVAo  Beta per SD (95% CI)  SD | 4.12 (3.00, 5.24)  39.21 | **<0.001** | -2.22x10^-2^ (-3.68x10^-2^, -7.64x10^-3^)  39.21 | **0.0029** |
| ET  Beta per SD (95% CI)  SD | 5.68 (4.50, 6.87)    49.98 | **<0.001** | -1.93x10^-2^ (-3.48x10^-2^, -3.78x10^-3^)  49.98 | **0.015** |
| AT  Beta per SD (95% CI)  SD | -0.79 (-2.17, 0.59)  48.23 | 0.26 | -1.42x10^-2^ (-3.23x10^-2^, 3.85x10^-3^)  48.23 | 0.12 |

AS: aortic stenosis, AT: acceleration Time, CI: confidence interval, ET: ejection Time, EOA: effective orifice area, SD: standard deviation, T-LVAo: time between left ventricular and aortic systolic pressure peaks

|  | Univariable | | Multivariable (N = 870) | | |
| --- | --- | --- | --- | --- | --- |
|  | **HR (95% CI)** | **p-value** | **HR (95% CI)** | **p-value** |  |
| Age | 1.00 (0.98, 1.02) | 0,99 |  |  |  |
| Sex (male) | 1.22 (0.95, 1.57) | 0,12 |  |  |  |
| Prior MI | 0.73 (0.50, 1.08) | 0,12 | 0.65 (0.41, 1.03) | 0.064 |  |
| Prior stroke | 1.32 (0.96, 1.83) | 0.088 |  |  |  |
| LVEF < 30% | 1.85 (1.32, 2.59) | <0.001 | 1.49 (0.99, 2.23) | 0.055 |  |
| Log(AVC Density) | 0.74 (0.63, 0.87) | <0.001 |  |  |  |
| sPAP > 55 mmHg | 1.40 (1.00, 1.96) | 0.047 |  |  |  |
| Diabetes on Insulin | 1.70 (1.26, 2.28) | <0.001 | 1.52 (1.07, 2.17) | 0.019 |  |
| Atrial fibrillation | 2.17 (1.68, 2.80) | <0.001 | 1.78 (1.30, 2.43) | <0.001 |  |
| COPD | 1.35 (0.98, 1.85) | 0.066 |  |  |  |
| GFR < 60 mL/min | 1.69 (1.29, 2.20) | <0.001 | 1.41 (1.02, 1.94) | 0.036 |  |
| P mean | 0.97 (0.96, 0.98) | <0.001 | 0.98 (0.97, 0.99) | 0.0069 |  |
| Stroke Volume Index | 0.96 (0.95, 0.98) | <0.001 |  |  |  |
| Heart rate (invasively derived) | 1.01 (1.00, 1.02) | 0.089 | 0.99 (0.98, 1.00) | 0.14 |  |
| EOA per BSA | 1.33 (0.36, 4.86) | 0,67 |  |  |  |
| T-LVAo > 52 ms (C_D_) | 0.85 (0.66, 1.09) | 0,2 | 1.07 (0.77, 1.49) | 0.67 |  |
| Ejection time > 274 ms (C_D_) | 0.43 (0.33, 0.55) | <0.001 | 0.61 (0.43, 0.87) | 0.0067 |  |
| Acceleration time > 158 ms (C_D_) | 0.68 (0.53, 0.87) | 0,0026 | 0.76 (0.52, 1.09) | 0.14 |  |

**Supplementary Table 3:** **Multivariable analysis for mortality with invasively derived T-LVAo, ET and AT for all patients**

AVC: aortic valve calcification, BSA: body surface area, COPD: chronic obstructive pulmonary disease, EOA: effective orifice area, GFR: glomerular filtration rate, HR: hazard ratio, LVEF: left ventricular ejection fraction, MI: myocardial infarction, sPAP: systolic pulmonary artery pressure

|  | Univariable | | Multivariable (N = 870) | | |
| --- | --- | --- | --- | --- | --- |
|  | **HR (95% CI)** | **p-value** | **HR (95% CI)** | **p-value** |  |
| Age | 1.04 (0.99, 1.09) | 0.082 | 1.07 (1.01, 1.13) | 0.028 |  |
| Sex (male) | 0.96 (0.53, 1.73) | 0,89 |  |  |  |
| Prior MI | 0.18 (0.02, 1.31) | 0.091 | 0.25 (0.03, 1.91) | 0.18 |  |
| Prior stroke | 2.15 (1.10, 4.23) | 0.026 | 1.81 (0.85, 3.83) | 0.12 |  |
| LVEF < 30% | 0.83 (0.20, 3.50) | 0.8 | 1.02 (0.23, 4.47) | 0.98 |  |
| Log(AVC Density) | 1.21 (0.71, 2.08) | 0.48 |  |  |  |
| sPAP > 55 mmHg | 2.05 (1.07, 3.96) | 0.032 |  |  |  |
| Diabetes on Insulin | 1.00 (0.39, 2.52) | 1 |  |  |  |
| Atrial fibrillation | 2.99 (1.64, 5.45) | <0.001 | 2.33 (1.25, 4.36) | 0.0077 |  |
| COPD | 1.48 (0.72, 3.07) | 0.29 |  |  |  |
| GFR < 60 mL/min | 1.30 (0.73, 2.31) | 0.37 |  |  |  |
| P mean | 0.98 (0.96, 1.01) | 0.2 |  |  |  |
| Stroke Volume Index | 0.97 (0.94, 0.99) | 0.019 |  |  |  |
| Heart rate (invasively derived) | 1.01 (0.99, 1.03) | 0.31 |  |  |  |
| EOA per BSA | 0.08 (0.00, 1.84) | 0.11 |  |  |  |
| T-LVAo > 52 ms (C_D_) | 0.60 (0.30, 1.21) | 0.16 | 0.52 (0.23, 1.19) | 0.12 |  |
| Ejection time > 274 ms (C_D_) | 0.29 (0.15, 0.53) | <0.001 | 0.52 (0.24, 1.12) | 0.096 |  |
| Acceleration time > 158 ms (C_D_) | 0.38 (0.21, 0.68) | 0.0011 | 0.42 (0.19, 0.92) | 0.029 |  |

**Supplementary Table 4:** **Multivariable analysis for mortality with invasively derived T-LVAo, ET and AT for HGAS-patients**

AVC: aortic valve calcification, BSA: body surface area, COPD: chronic obstructive pulmonary disease, EOA: effective orifice area, GFR: glomerular filtration rate, HGAS: high gradient aortic stenosis, HR: hazard ratio, LVEF: left ventricular ejection fraction, MI: myocardial infarction, sPAP: systolic pulmonary artery pressure

|  | Univariable | | Multivariable (N = 870) | |
| --- | --- | --- | --- | --- |
|  | **HR (95% CI)** | **p-value** | **HR (95% CI)** | **p-value** |
| Age | 0.99 (0.97, 1.01) | 0.46 |  |  |
| Sex (male) | 1.20 (0.91, 1.59) | 0.2 |  |  |
| Prior MI | 0.78 (0.52, 1.17) | 0.23 |  |  |
| Prior stroke | 1.14 (0.79, 1.64) | 0.49 |  |  |
| LVEF < 30% | 1.81 (1.27, 2.59) | 0.0012 |  |  |
| Log(AVC Density) | 0.72 (0.60, 0.87) | <0.001 |  |  |
| sPAP > 55 mmHg | 1.26 (0.85, 1.87) | 0.25 |  |  |
| Diabetes on Insulin | 1.71 (1.24, 2.35) | <0.001 | 1.57 (1.11, 2.23) | 0.012 |
| Atrial fibrillation | 1.89 (1.42, 2.51) | <0.001 | 1.56 (1.14, 2.14) | 0.0054 |
| COPD | 1.30 (0.91, 1.85) | 0.15 | 1.42 (0.96, 2.12) | 0.082 |
| GFR < 60 mL/min | 1.75 (1.29, 2.37) | <0.001 | 1.61 (1.14, 2.27) | 0.0064 |
| P mean | 0.94 (0.93, 0.96) | <0.001 | 0.95 (0.93, 0.98) | <0.001 |
| Stroke Volume Index | 0.97 (0.95, 0.99) | <0.001 |  |  |
| Heart rate (invasively derived) | 1.00 (1.00, 1.01) | 0.29 |  |  |
| EOA per BSA | 1.20 (0.27, 5.43) | 0.81 |  |  |
| T-LVAo > 52 ms (C_D_) | 0.91 (0.68, 1.22) | 0.53 | 0.99 (0.67, 1.45) | 0.95 |
| Ejection time > 274 ms (C_D_) | 0.47 (0.36, 0.62) | <0.001 | 0.62 (0.43, 0.88) | 0.0083 |
| Acceleration time > 158 ms (C_D_) | 0.77 (0.58, 1.02) | 0.066 | 0.91 (0.62, 1.34) | 0.63 |

**Supplementary Table 5:** **Multivariable analysis for mortality with invasively derived T-LVAo, ET and AT for LGAS-patients**

AVC: aortic valve calcification, BSA: body surface area, COPD: chronic obstructive pulmonary disease, EOA: effective orifice area, GFR: glomerular filtration rate, HR: hazard ratio, LGAS: low gradient aortic stenosis, LVEF: left ventricular ejection fraction, MI: myocardial infarction, sPAP: systolic pulmonary artery pressure

**Supplementary Figures:**

**
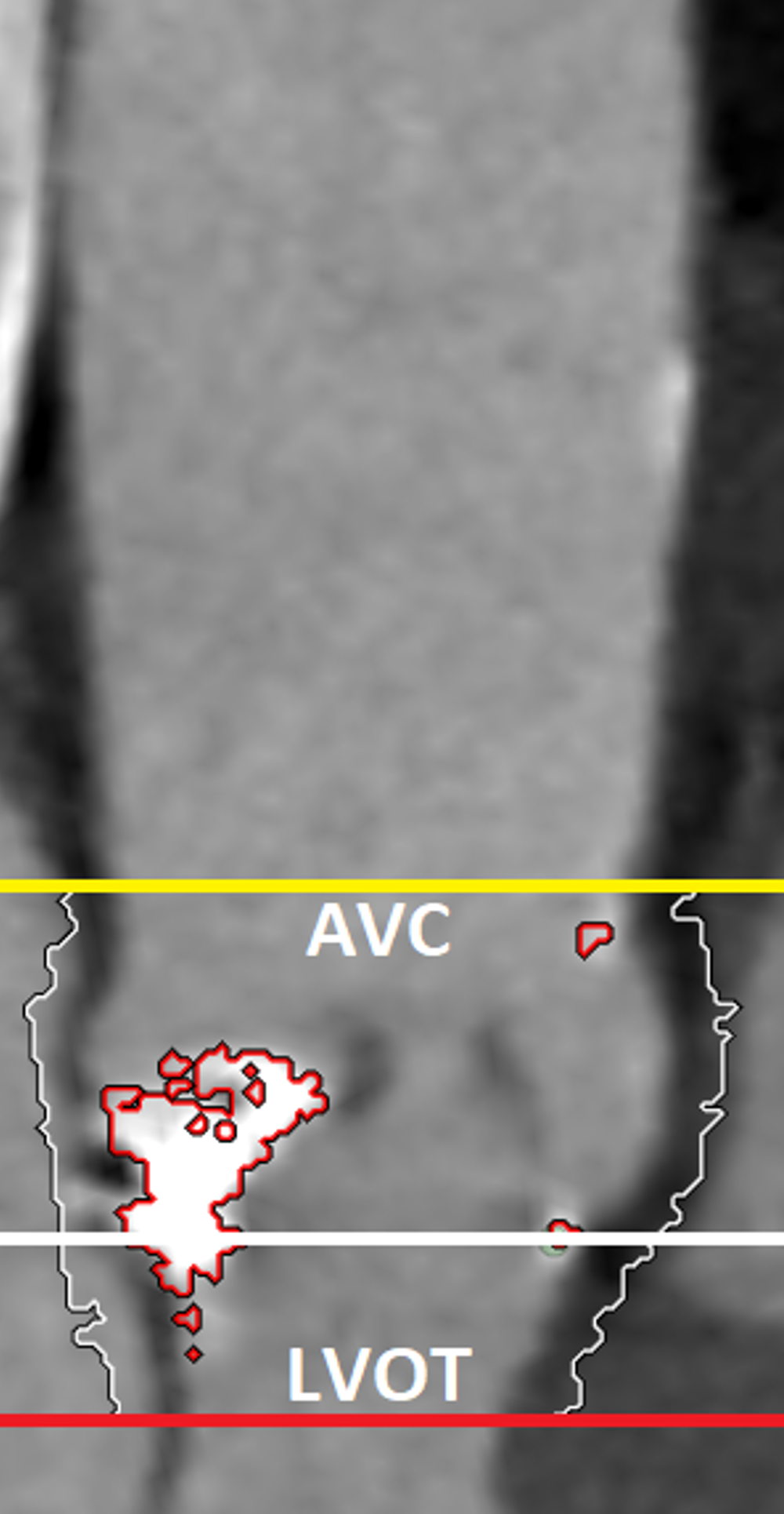
**

**Supplementary Figure 1: AVC and LVOT quantification in contrast-enhanced MDCT images**

Defined Areas with possible calcification**:** Annular plane (white line), AVC (white to yellow line), LVOT (white to red line).

AVC: aortic valve calcification, LVOT: left ventricular outflow tract, MDCT: multi-detector computed tomography
